# Early changes of fecal short‐chain fatty acid levels in patients with mild cognitive impairments

**DOI:** 10.1111/cns.14252

**Published:** 2023-05-05

**Authors:** Chao Gao, Binyin Li, Yixi He, Pai Huang, Juanjuan Du, Guiying He, Pingchen Zhang, Huidong Tang, Shengdi Chen

**Affiliations:** ^1^ Department of Neurology and Institute of Neurology, Ruijin Hospital Shanghai Jiao Tong University School of Medicine Shanghai China; ^2^ Lab for Translational Research of Neurodegenerative Diseases, Shanghai Institute for Advanced Immunochemical Studies (SIAIS) Shanghai Tech University Shanghai China

**Keywords:** biomarker, early diagnosis, mild cognitive impairment; fecal short‐chain fatty acids

## Abstract

**Aims:**

To compare the fecal levels of short‐chain fatty acids (SCFAs) in patients with mild cognitive impairment (MCI) and normal controls (NCs) and to examine whether fecal SCFAs could be used as the biomarker for the identification of patients with MCI. To examine the relationship between fecal SCFAs and amyloid‐β (Aβ) deposition in the brain.

**Methods:**

A cohort of 32 MCI patients, 23 Parkinson's disease (PD) patients, and 27 NC were recruited in our study. Fecal levels of SCFAs were measured using chromatography and mass spectrometry. Disease duration, ApoE genotype, body mass index, constipation, and diabetes were evaluated. To assess cognitive impairment, we used the Mini‐Mental Status Examination (MMSE). To assess brain atrophy, the degree of medial temporal atrophy (MTA score, Grade 0–4) was measured by structural MRI. Aβ positron emission tomography with ^18^F‐florbetapir (FBP) was performed in seven MCI patients at the time of stool sampling and in 28 MCI patients at an average of 12.3 ± 0.4 months from the time of stool sampling to detect and quantify Aβ deposition in the brain.

**Results:**

Compared with NC, MCI patients had significantly lower fecal levels of acetic acid, butyric acid, and caproic acid. Among fecal SCFAs, acetic acid performed the best in discriminating MCI from NC, achieved an AUC of 0.752 (*p* = 0.001, 95% CI: 0.628–0.876), specificity of 66.7%, and sensitivity of 75%. By combining fecal levels of acetic acid, butyric acid, and caproic acid, the diagnostic specificity was significantly improved, reaching 88.9%. To better verify the diagnostic performance of SCFAs, we randomly assigned 60% of participants into training dataset and 40% into testing dataset. Only acetic acid showed significantly difference between these two groups in the training dataset. Based on the fecal levels of acetic acid, we achieved the ROC curve. Next, the ROC curve was evaluated in the independent test data and 61.5% (8 in 13) of patients with MCI, and 72.7% (8 in 11) of NC could be identified correctly. Subgroup analysis showed that reduced fecal SCFAs in MCI group were negatively associated with Aβ deposition in cognition‐related brain regions.

**Conclusion:**

Reductions in fecal SCFAs were observed in patients with MCI compared with NC. Reduced fecal SCFAs were negatively associated with Aβ deposition in cognition‐related brain regions in MCI group. Our findings suggest that gut metabolite SCFAs have the potential to serve as early diagnostic biomarkers for distinguishing patients with MCI from NC and could serve as potential targets for preventing AD.

## Graphical abstract

Fecal acetic acid performed the best among SCFAs in distinguishing patients with MCI from NC. Combined fecal SCFAs increased the diagnostic specificity for identification of patients with MCI. Decreased fecal caproic acid in MCI patients showed the significantly negative correlation with Aβ deposition in several cognition‐related brain regions.

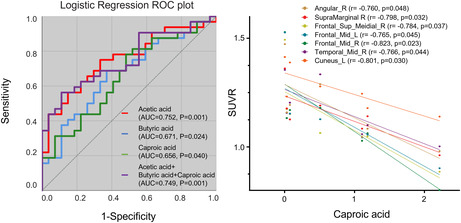

## 1 INTRODUCTION

Alzheimer's disease (AD) is the most common neurodegenerative disease and the most common pathological type of senile dementia, accounting for about 60%–70% of dementia cases. According to the World Alzheimer Report 2022, there were more than 55 million dementia patients worldwide in 2019, and the number is expected to rise to 139 million by 2050.^1^ The main pathological manifestations of AD include amyloid plaques formed by the deposition of amyloid‐β (Aβ) in the brain, and neurofibrillary tangles caused by abnormal aggregation of tau protein.^2^ However, the pathogenesis of AD and the causes of the above pathological changes need to be further clarified.

Emerging evidence showed specific gut microbial signature is closely associated with cognitive impairment^3^ and gut microbiota differed in patients with AD compared with the cognitively healthy status.^4^, ^5^ Importantly, this alteration in the gut microbiota occurred already in the pre‐onset stage mild cognitive impairment (MCI).^5^ In vivo studies found that the gut microbiota in transgenic mouse model of AD differed from that of healthy wild‐type (WT) mice. Transplantation of the fecal microbiota from WT mice into transgenic AD mice ameliorated the neuropathology and cognitive impairment.^6^ Short‐chain fatty acids (SCFAs) are important metabolites derived from the gut microbiota through fermentation of dietary fiber. SCFAs are absorbed into the mucous epithelium of the cecum and colon and some SCFAs enter the systemic circulation, thus SCFAs are speculated to be vital in gut–brain crosstalk.^7^, ^8^, ^9^ Studies have found that SCFAs improved cognition by reducing Aβ and tau pathology, regulating microglial functions, alleviating neuroinflammation, and increasing the expression of learning associated genes via histone deacetylase inhibition in rodent models in AD.^10^, ^11^

MCI is an intermediate state between normal cognitive aging and dementia and MCI is with great risk for the conversion to AD.^12^ Identification of patients with MCI and make early intervention are essential for preventing their progress to dementia, which is also the key to the success of clinical trials. In addition, treatment with SCFA (oral sodium butyrate) showed a dose‐dependent reduction in Aβ levels in the brains of AD mouse model at early disease stage.^7^ Thus, we aimed to analyze and compare the fecal levels of SCFAs in patients with MCI and normal controls to explore whether fecal SCFAs could serve as the early diagnostic biomarker to differentiate MCI patients from aging population. We also performed (PET) with ^18^F‐FBP some MCI patients to examine the relationship between fecal SCFAs and Aβ deposition in the brain to explore whether SCFAs could be potential targets for preventing AD from a therapeutic perspective.

## 2 METHODS

### 2.1 Standard protocol approvals, registrations, and patient consents

The study protocol was approved by the Ethics Committee of Ruijin Hospital, Shanghai Jiao Tong University School of Medicine. All participants signed written informed consent.

### 2.2 Participants and clinical evaluation

Thirty‐two patients with MCI and 27 NC were recruited from Ruijin Hospital, Shanghai Jiao Tong University School of Medicine. The diagnosis of MCI was based on the criteria of the National Institute on Aging‐Alzheimer's Association (NIA‐AA) workgroups.^13^ The global score of Clinical Dementia Rating (CDR) = 0.5 for MCI. Normal controls were recruited from community without cognitive problems. Demographic data included age, sex, years of education, body mass index (BMI), constipation, and diabetes. For MCI patients, clinical characteristics also included disease duration, ApoE genotype, Chinese version of mini–mental state examination (MMSE)^14^ and the degree of medial temporal atrophy (MTA score, Grade 0–4) measured by structural MRI. In addition, Aβ positron emission tomography with ^18^F‐FBP was performed in seven MCI patients at the time of stool sampling and in 28 MCI patients at an average of 12.3 ± 0.4 months from the time of stool sampling to detect and quantify Aβ deposition in the brain. In order to clarify the specificity of fecal SCFA expression level in different neurodegenerative diseases, 23 early PD patients were also recruited in our study. The diagnosis of PD was according to the Movement Disorder Society (MDS) PD criteria.^15^ Exclusion criteria included evidence of stroke, or other neurodegenerative causes of dementia. Participants were also excluded if they had a history of irritable bowel syndrome, inflammatory bowel disease, colitis, colon cancer, or use of antibiotics or probiotic supplements within 3 months of enrollment.

### 2.3 Fecal sample collection and measurement of fecal levels of SCFAs

Participants were asked to collect a fecal sample in the morning using insulated fecal collection containers with surrounding ice. After arriving at the laboratory, it was stored at −80°C prior to progressing. The analysis of short‐chain fatty acids was performed following routine operations by Tinygene Bio‐Tech (Shanghai) Co., Ltd. Take 50 mg of feces in the 1.5 mL centrifugal tube, add 500 μL of water, add 100 mg of glass beads, 1 min for homogenate, centrifuge for 10 min at 4°C with 13,200 *g*, and then 200 μL supernatant was collected. Next, add 100 μL 15% phosphate, 20 μL 375 μg/mL internal standard (4‐methylic acid) solution and 280 μL ether, 1 min for homogenate, centrifuge for 10 min at 4°C with 12,000 rpm, and the supernatant was collected and used to detect SCFAs. Concentrations of SCFAs were determined using gas chromatography–mass spectrometry (GC–MS) with Thermo Trace 1300 and Thermo ISQ 7000 under the full scan and SIM mode. For targeted fecal metabolomic detection, internal standard references of amino acids and related metabolites were purchased from Cambridge Isotope Laboratories (US), and the methods of metabolite extraction, instrument, and data analyzing of target metabolomic detection were conducted as previously described^16^ with modification.

### 2.4 APOE genotyping

Genomic DNA was extracted from peripheral blood through standardized phenol/chloroform extraction method. Genotyping analysis of APOE was performed as previously described.^17^

### 2.5 Data splitting into training dataset and testing dataset

To better verify the diagnostic performance of SCFAs, we randomly split our data into training dataset and testing dataset. Thirty‐two patients with MCI and 27 NC were recruited in our study. We randomly chose 60% of the participants (MCI: 19, NC: 16) for training, and the remaining 40% (MCI: 13, NC: 11) were used for independent testing. In the training dataset, we ensured that the age and gender between MCI and NC groups were matched.

### 2.6 Statistical analysis

Continuous variables were expressed as mean ± SD and categorical variables as numbers and percentages. The Kolmogorov–Smirnov normality test examines if variables are normally distributed. Comparisons of demographic and clinical data between the MCI and NC groups were performed by independent sample *t*‐test or Mann–Whitney *U* test or for quantitative, and the Chi‐square test for categorical variables, respectively. Partial least squares‐discrimination analysis (PLS‐DA) was performed using R 4.2.1. Permutational multivariate analysis of variance (PERMANOVA) was used for PLS‐DA using Bray–Curtis distance matrices. The levels of SCFAs in feces were log 10‐transformed to achieve normal distribution before further analysis. But even after log‐transformation for SCFAs, the levels of some of the log 10‐transformed SCFAs still showed non‐Gaussian distribution in the Kolmogorov–Smirnov test, so the differences between the MCI and NC groups were assessed using the Mann–Whitney *U* test or independent sample *t*‐test. We used the area under the receiver operating characteristic (ROC) curve (AUC) to quantify the model's diagnostic performance for exploring the ability of SCFAs to distinguish between patients with MCI and NC. Seven MCI patients who performed PET were underwent subgroup analysis. The normalized PET scans were smoothed using a Gaussian filter of 12 mm full‐width at half‐maximum. Using the whole cerebellum as reference region, standard uptake value ratio (SUVR) images were computed utilizing in‐house scripts in MATLAB R2014b (The MathWorks, Inc.). The correlation between fecal levels of SCFAs and SUVR were analyzed to evaluate the effect of fecal SCFAs on Aβ deposition. To examine the associations between SCFAs and clinical characteristics, Spearman rank correlation analysis was performed.

## 3 RESULTS

### 3.1 Demographics and clinical characteristics of MCI patients and normal controls

Thirty‐two patients with MCI (65.4 ± 7.3 years of age, 43.8% male), and 27 NC (63.6 ± 5.4 years of age, 44.4% male) were enrolled in this study (Table 1). The demographic and clinical characteristics of patients with MCI and NC were of no significant differences in age, gender, BMI, diabetes, constipation, and years of education (Table 1). As expected, patients with MCI had a lower MMSE score than patients with NC (26.9 ± 1.5 vs. 29.1 ± 1.3, respectively, *p* = 0.000, Table 1). Patients with MCI had disease duration of 2.3 ± 2.3 years (from the onset of cognitive impairment) and did not take any acetylcholinesterase inhibitors and/or memantine. For ApoE genotype, among the 32 MCI patients, 6 (18.7%) carried ε2/ε3, 15 (46.9%) carried ε3/3, 7 (21.9%) carried ε3/ε4 and 4 (12.5%) carried ε4/ε4. For MTA grade, of the 32 MCI patients, 8 (25.0%) are in grade 0, 14 (43.8%) are in grade 1, 4 (12.5%) are in grade 2 and 6 (18.7%) are in grade 3.

**TABLE 1:** Clinical and demographic data of patients with mild cognitive impairment and cognitively normal controls.

Characteristics	MCI patients (*n* = 29)	Normal controls (*n* = 27)	*p* Value
Age, years	65.4 ± 7.3	63.6 ± 5.4	0.410
Male, *n* (%)	14 (43.8)	12 (44.4)	0.957
Education, years	12.3 ± 2.8	11.9 ± 2.9	0.785
BMI	23.0 ± 2.9	24.2 ± 3.0	0.134
MMSE score	26.9 ± 1.5	29.1 ± 1.3	0.000**
Constipation (%)	1 (3.1)	3 (11.1)	0.486
Diabetes (%)	3 (9.4)	1 (3.7)	0.778
Disease duration, years	2.3 ± 2.3		
ApoE genotype			
ε2/ε3	6 (18.7)		
ε3/ε3	15 (46.9)		
ε3/ε4	7 (21.9)		
ε4/ε4	4 (12.5)		
MTA, grade			
0	8 (25.0)		
1	14 (43.8)		
2	4 (12.5)		
3	6 (18.7)		

*Note*: Age, education, BMI, and MMSE scores, and disease duration are expressed as means ± standard deviation. Gender, constipation, diabetes, ApoE genotype, and MTA grade are expressed as a proportion; Differences between groups were assessed using the Chi‐square test for categorical data and Mann–Whitney *U* test or independent sample *t*‐test for numerical data.

Abbreviations: BMI, body mass index; MCI, mild cognitive impairment; MMSE, mini–mental state examination; MTA, medial temporal atrophy.

**p < 0.01.

### 3.2 Comparison of fecal levels of different types of SCFAs between patients with MCI and normal controls and diagnostic model construction

**FIGURE 1:**
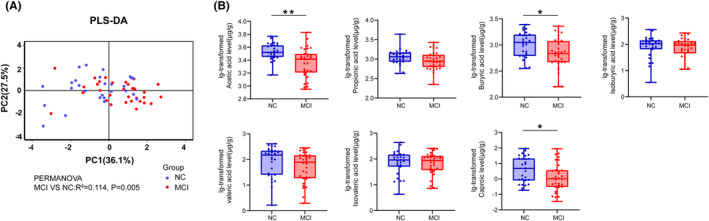
Comparison of fecal levels of short chain fatty acids in patients with MCI and normal controls. (A) Partial least squares‐discrimination analysis (PLS‐DA) of fecal SCFAs in patients with MCI and NC. (B) Boxplot shows median with range for fecal levels of acetic acid, propionic acid, butyric acid, isobutyric acid, valeric acid, isovaleric acid, and caproic acid in patients with mild cognitive impairment (MCI) and normal controls. *p* Values were calculated using Mann–Whitney U test or independent sample *t*‐test. MCI, mild cognitive impairment; NC, normal controls. *p < 0.05, **p < 0.01.

**FIGURE 2:**
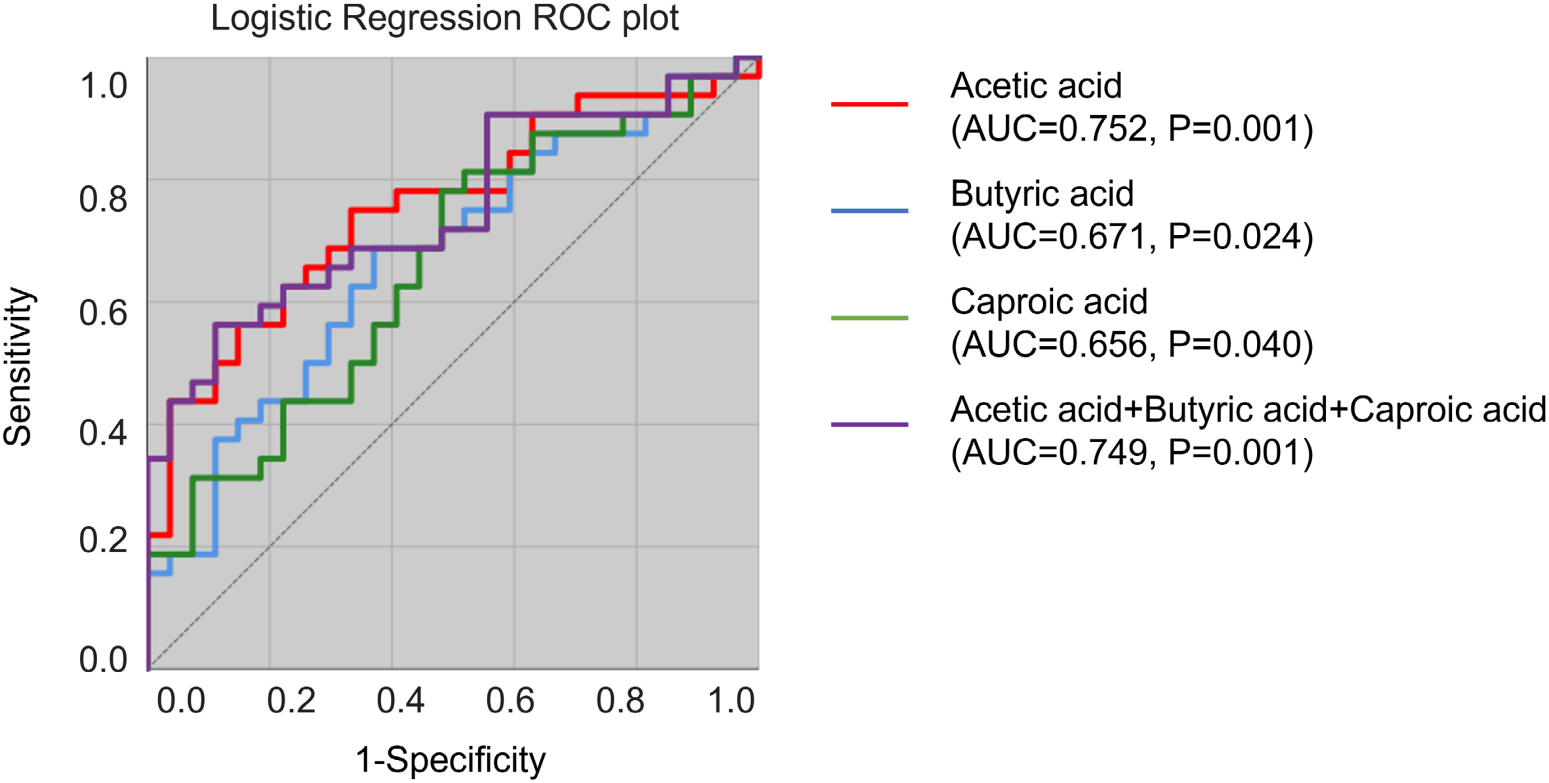
The differential models of the receiver operating characteristic (ROC) analysis distinguishing patients with MCI and normal controls based on the fecal levels of acetic acid, butyric acid, and caproic acid.

The PLS‐DA analysis of fecal SCFAs indicated a distinct profile between patients with MCI and NC (PERMANOVA: *R* ^2^ = 0.114, *p* = 0.005) (Figure 1A). In the comparison of fecal SCFAs, patients with MCI had significantly lower fecal levels of acetic acid (*p* = 0.001), butyric acid (*p* = 0.024), and caproic acid (*p* = 0.040) than NC (Figure 1B and Table 2). Based on the results that patients with MCI had significantly lower fecal levels of acetic acid, butyric acid, and caproic acid than NC, we next performed ROC curve analysis to predict MCI occurrence. Acetic acid performed the best in discriminating MCI from NC compared with butyric acid and caproic acid, achieved an AUC of 0.752 (*p* = 0.001, 95% CI: 0.628–0.876), specificity of 66.7% and sensitivity of 75%. Importantly, we found that when acetic acid, butyric acid, and caproic acid were combined for the differential diagnosis of MCI and NC, the specificity was significantly improved, reaching 88.9%, although the AUC was similar to that of acetic acid (AUC = 0.749, *p* = 0.001, 95% CI: 0.625–0.873) (Figure 2).

**TABLE 2:** SCFA levels in patients with mild cognitive impairment and normal controls.

	MCI patients (*n* = 32)	Normal controls (*n* = 27)	*p* Value
Acetic acid	2609.53 ± 1320.21	3573.08 ± 1001.30	0.001**
Propionic acid	1052.19 ± 570.46	1251.59 ± 710.13	0.091
Butyric acid	825.28 ± 544.49	1156.32 ± 583.59	0.024*
Isobutyric acid	96.60 ± 65.88	112.94 ± 82.9	0.438
Valeric acid	93.36 ± 83.23	145.77 ± 118.39	0.107
Isovaleric acid	96.29 ± 72.44	110.15 ± 98.72	0.738
Caproic acid	8.39 ± 18.65	15.98 ± 22.66	0.040*

*Note*: SCFA levels (μg/g) are expressed as means ± standard deviation. Differences between groups were assessed using Mann–Whitney *U* test or independent samples *t*‐test.

Abbreviation: MCI, mild cognitive impairment.

**p < 0.01, *p < 0.05.

In order to better verify the diagnostic performance of SCFAs, we randomly split our data into training dataset and testing dataset. The entire dataset in our study had 59 recordings (MCI: 32, NC: 27), in which 60% of the recordings (MCI: 19, NC: 16) were used for training, whereas the remaining 40% (MCI: 13, NC: 11) were used for independent testing. In the training dataset, we ensured that the age and gender between MCI and NC groups were matched. We first compared fecal SCFAs levels between 19 MCI patients and 16 NC in the training dataset. Results showed that only acetic acid showed significantly difference between these two groups (MCI vs. NC: 2492.29 ± 1032.23 vs. 3632.60 ± 1040.40, *p* = 0.003). Based on the fecal levels of acetic acid, ROC curve was constructed, and we achieved an AUC of 0.757 (95% CI: 0.598–0.915) to distinguish patients with MCI from NC. Next, the ROC curve was evaluated in the independent test data and with the cutoff value of 2692.84 μg/g, 61.5% (8 in 13) of patients with MCI, and 72.7% (8 in 11) of NC could be identified correctly.

### 3.3 The association of fecal SCFAs levels and amyloid‐β deposition in MCI group

**FIGURE 3:**
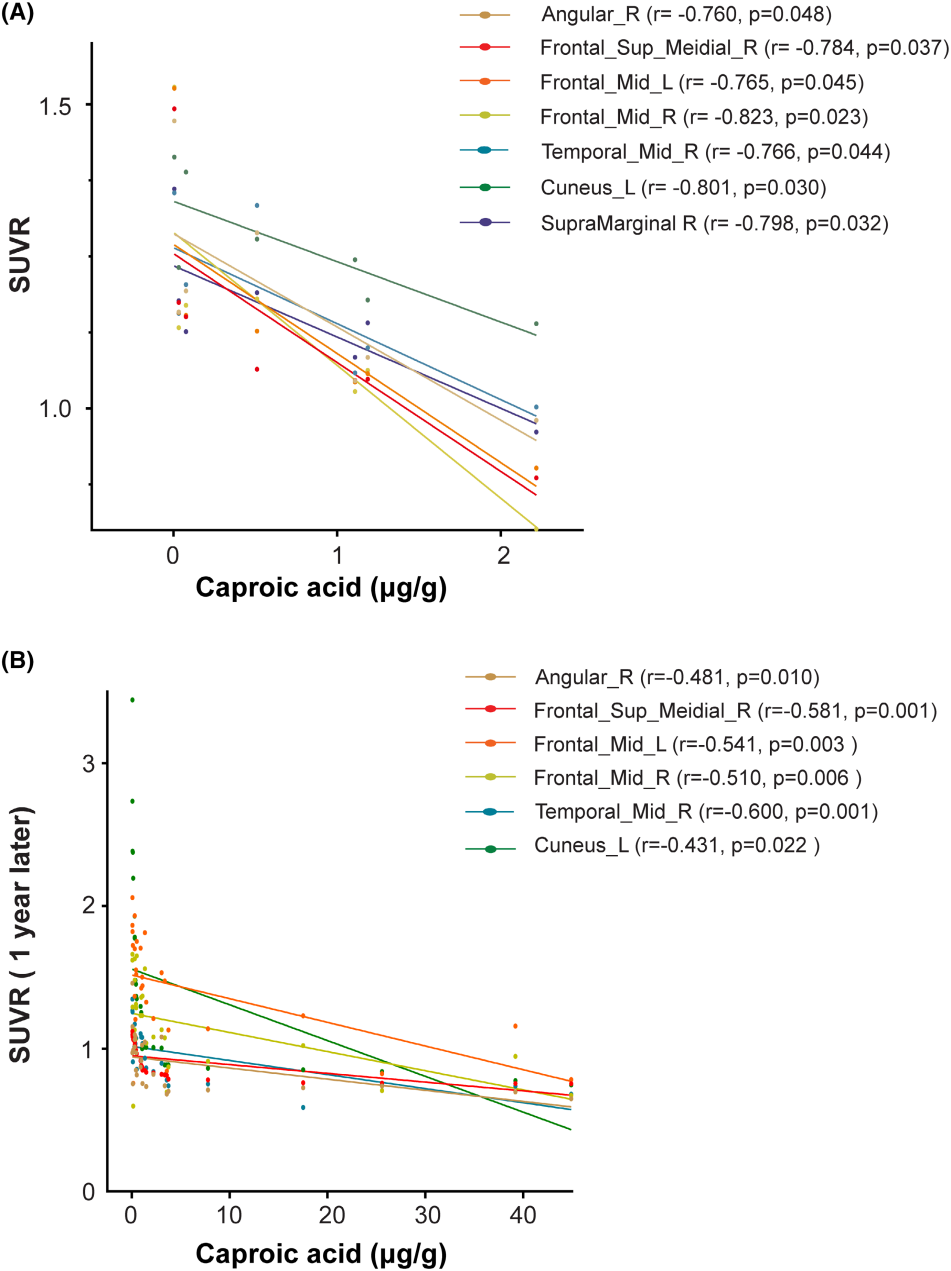
Fecal levels of caproic acid negatively correlated with amyloid‐β deposition (SUVR) in several cognition‐related brain regions in MCI patients. (A) Seven MCI patients were administered with amyloid‐β positron emission tomography inspection with ^18^F‐florbetapir (FBP). (B) Twenty‐eight MCI patients were recalled to complete Aβ PET with ^18^F‐FBP at an average of 12.3 ± 0.4 months from the time of stool sampling. Amyloid deposition was measured by standard uptake value ratio (SUVR) with reference to cerebellum. Mid, middle; L, left; R, right; Angular: Angular gyrus; Cuneus: Cuneus gyrus; Frontal_Sup_Medial: Superior frontal gyrus, medial; SupraMarginal: supramarginal gyrus.

Seven MCI patients were administrated Aβ PET with ^18^F‐FBP at the time of stool sampling to detect and quantify Aβ deposition in the brain. We found the significantly negative correlation between fecal levels of caproic acid and SUVR of Aβ in several brain areas including frontal lobe, temporal lobe, angular gyrus, and cuneus gyrus (*r*: −0.760 to −0.823, *p* < 0.05) (Figure 3A, Table S1). Acetic acid, propionic acid, and butyric acid also showed the significantly negative relationship with SUVR of Aβ in supplementary motor area (*r* = −0.821, *p* = 0.024; *r* = −0.806, *p* = 0.029; *r* = −0.814, *p* = 0.026, respectively). In order to further confirm the correlation between the caproic acid level in feces and the deposition of Aβ in the brain. Twenty‐eight MCI patients were recalled to complete Aβ PET with ^18^F‐FBP at an average of 12.3 ± 0.4 months from the time of stool sampling. Correlation analysis revealed that fecal caproic acid levels were still significantly negatively correlated with Aβ deposition in these brain regions (*r*: −0.431 ~ −0.600, *p* < 0.05) (Figure 3B).

### 3.4 The association of fecal SCFAs levels and clinical characteristics in MCI group

To examine the associations between fecal SCFAs and clinical characteristics including age, cognitive function measured by MMSE, ApoE genotype, disease duration, and hippocampus atrophy measured by MTA score, Spearman rank correlation between the altered fecal SCFAs and clinical characteristics was performed. But only acetic acid showed negative correlation with age (*r* = −0.387, *p* = 0.029) in MCI group (Figure S1, Table S2).

### 3.5 Comparison of fecal SCFA expression profiles between MCI and PD patients

**FIGURE 4:**
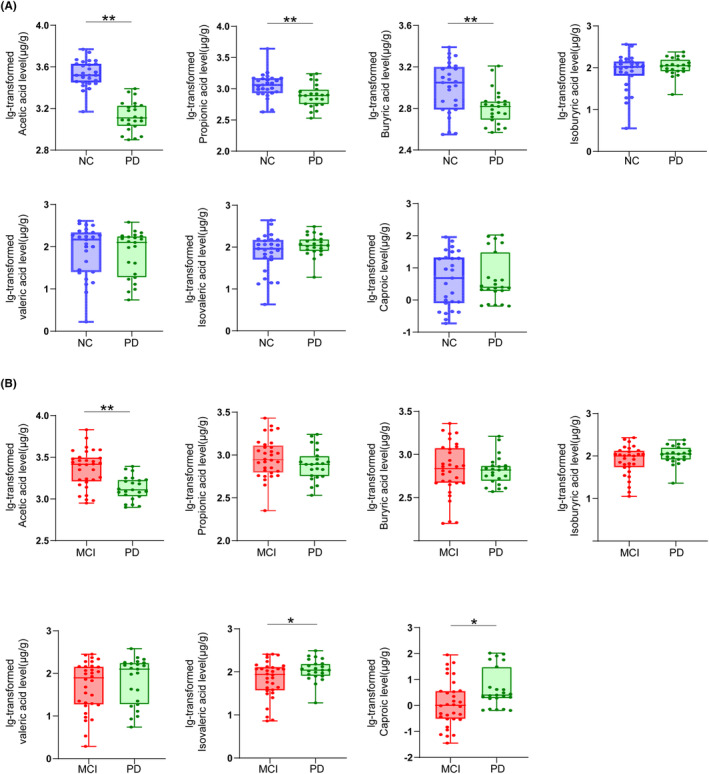
Comparisons of fecal levels of short‐chain fatty acids between PD patients and normal controls, and between PD patients and MCI patients. Boxplot shows median with range for fecal levels of acetic acid, propionic acid, butyric acid, isobutyric acid, valeric acid, isovaleric acid, and caproic acid in patients with PD and normal controls (A) and in patients with MCI and PD (B). *p* Values were calculated using Mann–Whitney *U* test or independent sample *t*‐test. MCI, mild cognitive impairment; NCs, normal controls; PD, Parkinson's disease. *p < 0.05, **p < 0.01.

In order to clarify the specificity of fecal SCFA expression level in different neurodegenerative diseases, we also detected SCFAs in feces of early PD patients (*n* = 23, H‐Y stage 1.5 ± 0.5; disease duration 1.8 ± 1.8 years) in this study. The demographic and clinical characteristics of patients with PD and NC or PD and MCI were of no significant differences in age, gender, BMI, and MMSE score (Table S3). First, we found that patients with PD had significantly lower fecal levels of acetic acid (*p* = 0.000), propionic acid (*p* = 0.004), and butyric acids (*p* = 0.002) than NC (*n* = 27) (Figure 4A), which is consistent with previous reports.^18^, ^19^ Next, we compared the SCFAs level in 23 PD patients and 32 MCI patients and found that acetic acid (*p* = 0.000), isovaleric acid (*p* = 0.020), and caproic acid (*p* = 0.011) were significantly different between the two groups, which suggested that the expression profile of fecal SCFAs is different in MCI and PD patients (Figure 4B).

## 4 DISCUSSION

In this study, we found that patients with MCI had lower fecal levels of SCFAs (acetic acid, butyric acid, and caproic acid) compared with NC. Acetic acid performed the best among SCFAs in distinguishing patients with MCI from NC. Combined fecal SCFAs (acetic acid + butyric acid + caproic acid) could increase the diagnostic specificity for identification of patients with MCI. We further randomly split our data into training dataset and testing dataset to better verify the diagnostic performance of SCFAs and found that using the diagnostic model constructed with acetic acid, 61.5% (8 in 13) of patients with MCI, and 72.7% (8 in 11) of NC could be identified correctly. Importantly, we found that the decreased fecal level of caproic acid in MCI patients showed the significantly negative correlation with SUVR of Aβ in several cognition‐related brain regions.

MCI represents a transitional state between normal aging and dementia. Approximately 10–15% of individuals with MCI develop dementia‐usually AD every year, compared with 1%–2% in aging populations with cognitively healthy status.^20^, ^21^ MCI is with great risk for the conversion to AD^12^ and could be considered as a prodromal phase of AD. Identification of patients with MCI and make early intervention are essential for preventing their progress to dementia, which is also the key to the success of clinical trials. Thus, exploring early diagnostic biomarkers to detect patients with MCI from aging population is urgently needed. In the past decades, researchers have been focusing on exploring imaging, cerebrospinal fluid (CSF) and blood biomarkers for early diagnosis of AD.^22^, ^23^, ^24^, ^25^, ^26^ In fact, stool is more accessible and noninvasive sample source compared with above samples. In recent years, as the brain–gut axis plays a prominent role in the pathogenesis of neurodegenerative diseases,^7^, ^8^, ^9^, ^27^ the expression level of gut microbiota in the progress of neurodegenerative diseases such as AD and PD have been examined.^5^, ^18^, ^28^ Our previous study identified differences of gut microbiota between MCI and NC. Using the diagnostic model with all different genera input, 93% (28 in 30) of patients with MCI could be identified correctly.^5^ In our study, we further examined the levels of SCFAs, the metabolites derived from the gut microbiota, in fecal samples in patients with MCI, NC, and PD. We found that patients with MCI had significantly lower fecal levels of acetic acid, butyric acid, and caproic acid than NC and expression profile of fecal SCFAs is different in MCI and PD patients. Among fecal SCFAs, acetic acid performed the best in discriminating MCI from NC. By combining fecal levels of acetic acid, butyric acid, and caproic acid, the diagnostic specificity was significantly improved, reaching 88.9%. After we randomly assigned 60% of participants into training dataset and 40% into testing dataset, only acetic acid showed significantly difference between MCI and NC group in the training dataset. Based on the fecal levels of acetic acid, ROC curve was constructed, and we found using this diagnostic model 61.5% (8 in 13) of patients with MCI, and 72.7% (8 in 11) of NC could be identified correctly in the independent test data. Although the accuracy of using SCFAs in the diagnosis of MCI needs to be further improved, we have already detected the changes of SCFAs in the MCI patients. In the future, we can expand the sample size and utilize SCFAs combined with other metabolites derived from the gut microbiota for joint diagnosis, which will facilitate the identification of patients with MCI.

SCFAs are the main bacterial products of dietary fibers and resistant starches through fermentation by the microbiota in the cecum and colon.^29^ SCFAs are involved in a series of physiological progresses via interactions with G protein‐coupled receptors or histone deacetylases in the human body, such as immune regulation and host metabolism.^10^, ^30^ SCFAs are also important mediators of gut–brain interactions and participate in the occurrence and development of many neurodegenerative diseases, including AD and PD.^7^, ^8^, ^9^, ^10^, ^11^, ^31^ Emerging evidence has shown that SCFAs modulate the neuropathological progresses underlying AD. Besides, SCFAs are indispensable for the maturation of microglia and have important regulatory effects on immune homeostasis. For example, acetate impacts microglial cells and reduces blood–brain barrier permeability.^32^, ^33^ Acetate supplementation can inhibit neuroinflammation via ERK/JNK/NF‐κB pathway in an AD mouse model.^34^ Sodium butyrate administration ameliorated cognitive impairment by increasing expression of learning associated genes and reducing Aβ and tau pathologies in AD mouse models.^7^, ^8^, ^35^, ^36^ Therefore, the above evidence suggests that SCFAs play a protective role in the pathogenesis of AD and supplement the gut with SCFAs may help in preventing or ameliorating AD pathology. In our study, we found that reduced levels of acetic acid, butyric acid, and caproic acid in the MCI group, to some extent, may weaken the protective effect of SCFAs and promoted the cognitive impairment.

An important finding of this study was that reduced levels of SCFAs in the MCI group were negatively associated with the Aβ deposition in the brain. Fecal levels of caproic acid significantly and negatively correlated with SUVR of Aβ in several brain areas including frontal lobe, temporal lobe, angular gyrus, and cuneus gyrus. Besides, acetic acid and butyric acid showed the significantly negative relationship between SUVR of Aβ in supplementary motor area. Studies have shown that angular gyrus is involved in a variety of cognitive processes.^37^ The cuneus is involved early in cognitive impairment. Cuneus atrophy occurs even before the episodic memory loss and the atrophy of cuneus is associated with an increased risk of AD.^38^ Frontal lobe dysfunctions have been found in subjects with MCI,^39^ and temporal lobe is the earliest brain region with pathological changes in MCI. MCI showed lesser activation in supplementary motor area, commonly associated with motor preparation and planning.^40^ Supplementary motor area damage also affects working memory.^41^ The above evidence indicated that in the MCI group, patients with reduced fecal SCFAs had increased Aβ deposition in cognition‐related brain regions. One previous study found that oral sodium butyrate administration showed a dose‐dependent reduction in Aβ levels in the brains of AD mouse model at the early stage of disease progression.^7^ These evidence and our findings together suggest that SCFAs could serve as potential targets for preventing AD.

Our study has several limitations. First, blood SCFA concentrations and gut microbiota were not measured, so we did not compare plasma and fecal levels of SCFAs in patients with MCI and NC to delineate the correlation of these levels and link to changes in gut microbiota. Second, our cohort may be limited in sample size to find statistically robust differences in SCFAs. In our study, the levels of all SCFAs in the MCI group were lower than those in the NC group, although only acetic acid, butyric acid, and caproic acid showed a statistical difference between the two groups. If the sample size was expanded, it was expected that more SCFAs would have a statistical difference between the two groups, which may further improve the accuracy of early diagnosis by combining more SCFAs. Meanwhile, it is more conducive for us to divide the samples into training datasets and testing datasets to independently verify the diagnostic model. Third, the cross‐sectional studies cannot reflect the dynamic changes of SCFAs and their causal relationship with the disease process. Further longitudinal follow‐up studies are needed to serially measure both fecal and plasma SCFAs levels.

## 5 CONCLUSION

Our findings suggest that gut metabolite SCFAs have the potential to serve as early diagnostic biomarkers for distinguishing patients with MCI from normal controls. Among fecal SCFAs, the level of acetic acid changed most significantly at early disease stage and acetic acid performed the best in the differential diagnosis of MCI from normal controls. Reduced fecal SCFAs were negatively associated with Aβ deposition in cognition‐related brain regions in MCI group, suggesting that SCFAs could serve as potential targets for preventing AD.

## FUNDING INFORMATION

This work was supported by grants from the National Natural Science Foundation of China (82171401, 82101477, 82271441), Shanghai Municipal Science and Technology Major Project (2018SHZDZX05), and Shanghai Rising‐Star Program (21QA1405800).

## CONFLICT OF INTEREST STATEMENT

The authors declare no conflict of interest.

## Supporting information


Data S1

## Data Availability

The data supporting the findings of this study are available on request from the corresponding author.
