# Supplementary material for: Early changes of fecal short‐chain fatty acid levels in patients with mild cognitive impairments
Source: CNS Neurosci Ther. 2023 May 5;29(11):3657–66. doi: 10.1111/cns.14252 (PMC10580335; doi:10.1111/cns.14252)
Supplement: Supplementary file 1 — Data S1 [file CNS-29-3657-s001.docx]

**Supplementary Table 1. Fecal levels of short chain fatty acids negatively correlated with amyloid-β deposition (SUVR) in MCI patients**

|  | MCI-1 | MCI-2 | MCI-3 | MCI-4 | MCI-5 | MCI-6 | MCI-7 | Caproic acid | |
| --- | --- | --- | --- | --- | --- | --- | --- | --- | --- |
|  |  |  |  |  |  |  |  | r | p |
| Angular_R | 0.980 | 1.289 | 1.193 | 1.473 | 1.045 | 1.084 | 1.159 | -0.760 | 0.048 |
| Cuneus_L | 1.139 | 1.278 | 1.389 | 1.413 | 1.244 | 1.178 | 1.232 | -0.801 | 0.030 |
| Frontal_Mid_L | 0.902 | 1.127 | 1.153 | 1.526 | 1.043 | 1.057 | 1.158 | -0.765 | 0.045 |
| Frontal_Mid_R | 0.802 | 1.180 | 1.169 | 1.528 | 1.028 | 1.063 | 1.133 | -0.823 | 0.023 |
| Frontal_Sup_Medial_R | 0.886 | 1.064 | 1.151 | 1.493 | 1.046 | 1.048 | 1.174 | -0.784 | 0.037 |
| SupraMarginal_R | 0.961 | 1.190 | 1.126 | 1.361 | 1.084 | 1.141 | 1.177 | -0.798 | 0.032 |
| Temporal_Mid_R | 1.002 | 1.334 | 1.204 | 1.354 | 1.058 | 1.100 | 1.156 | -0.766 | 0.044 |
| Supp_Motor_Area_L* | 1.118 | 1.144 | 1.258 | 1.591 | 1.309 | 1.115 | 1.257 |  |  |

Seven MCI patients were administered with amyloid-β positron emission tomography inspection with ^18^F-florbetapir (FBP). Amyloid deposition was measured by standard uptake value ratio (SUVR) with reference to cerebellum.

Asterisk: the significantly negative relationship between SUVR of amyloid and fecal levels of acetic acid (r= -0.821, p= 0.024) or propionic acid (r= -0.806, p= 0.029) or butyric acid (r=-0.814, p= 0.026).

Mid, middle; L, left; R, right; Angular: Angular gyrus; Cuneus: Cuneus gyrus; Frontal_Sup_Medial: Superior frontal gyrus, medial; SupraMarginal: supramarginal gyrus; Supp_Motor_Area: Supplementary motor area.

**Supplementary Table 2. Correlation between Fecal Levels of Short Chain Fatty Acids of patients with MCI and clinical characteristics**

|  | Age | | MMSE | | Disease duration | | ApoE | | MTA | |
| --- | --- | --- | --- | --- | --- | --- | --- | --- | --- | --- |
|  | r | p | r | p | r | p | r | p | r | p |
| Acetic acid | -0.387 | 0.029* | -0.089 | 0.630 | 0.124 | 0.497 | -0.026 | 0.888 | -0.271 | 0.140 |
| Propionic acid | -0.278 | 0.124 | -0.053 | 0.771 | 0.023 | 0.902 | -0.320 | 0.074 | -0.205 | 0.269 |
| Butyric acid | -0.231 | 0.204 | -0.130 | 0.478 | -0.032 | 0.862 | -0.082 | 0.656 | -0.224 | 0.226 |
| Isobutyric acid | 0.019 | 0.917 | -0.104 | 0.572 | 0.039 | 0.834 | 0.061 | 0.741 | -0.104 | 0.579 |
| Valeric acid | 0.074 | 0.687 | -0.177 | 0.332 | 0.056 | 0.762 | 0.159 | 0.385 | -0.034 | 0.855 |
| Isovaleric acid | 0.051 | 0.783 | -0.082 | 0.654 | -0.003 | 0.985 | 0.058 | 0.751 | -0.098 | 0.599 |
| Caproic acid | 0.089 | 0.627 | -0.103 | 0.574 | 0.111 | 0.544 | 0.106 | 0.562 | 0.144 | 0.439 |

Spearman rank correlation analysis was performed to examine the associations between SCFAs and clinical characteristics.

Abbreviations: MMSE, Mini–Mental State Examination; MTA, medial temporal atrophy; r, correlation coefficient. *p<0.05.

**Supplementary table 3. Clinical and demographic data of patients with mild cognitive impairment, Parkinson’s disease, and cognitively normal controls**

| **Characteristics** | **MCI patients（n=29）** | **PD patients（n=23）** | **Normal controls** | **P value**  **MCI vs. PD** | **P value**  **PD vs. NC** |
| --- | --- | --- | --- | --- | --- |
| Age, years | 65.4 ± 7.3 | 63.1 ± 2.0 | 63.6 ± 5.4 | 0.308 | 0.852 |
| Male, n (%) | 14 (43.8) | 11 (47.8) | 12 (44.4) | 0.765 | 0.811 |
| BMI | 23.0 ± 2.9 | 23.6 ± 1.6 | 24.2 ± 3.0 | 0.334 | 0.424 |
| MMSE score | 26.9 ± 1.5 | 28.2 ± 2.3 | 29.1 ± 1.3 | 0.000^**^ | 0.102 |
| Disease duration, years | 2.3 ± 2.3 | 1.8 ± 1.8 |  | 0.277 |  |
| H-Y stage |  | 1.5 ± 0.5 |  |  |  |

NOTE. Age, BMI, and MMSE scores, and disease duration are expressed as means ± standard deviation. Gender is expressed as a proportion; Differences between groups were assessed using the Chi-square test for categorical data and Mann-Whitney U test or Independent Samples t-test for numerical data.

Abbreviations: MCI, mild cognitive impairment; PD, Parkinson’s disease; BMI, body mass index; MMSE, Mini–Mental State Examination; H-Y. Hoehn-Yahr.

**p < 0.01

**Supplementary Figure 1.** **Correlation between fecal levels of** **short chain fatty acids of patients with MCI and clinical characteristics.**


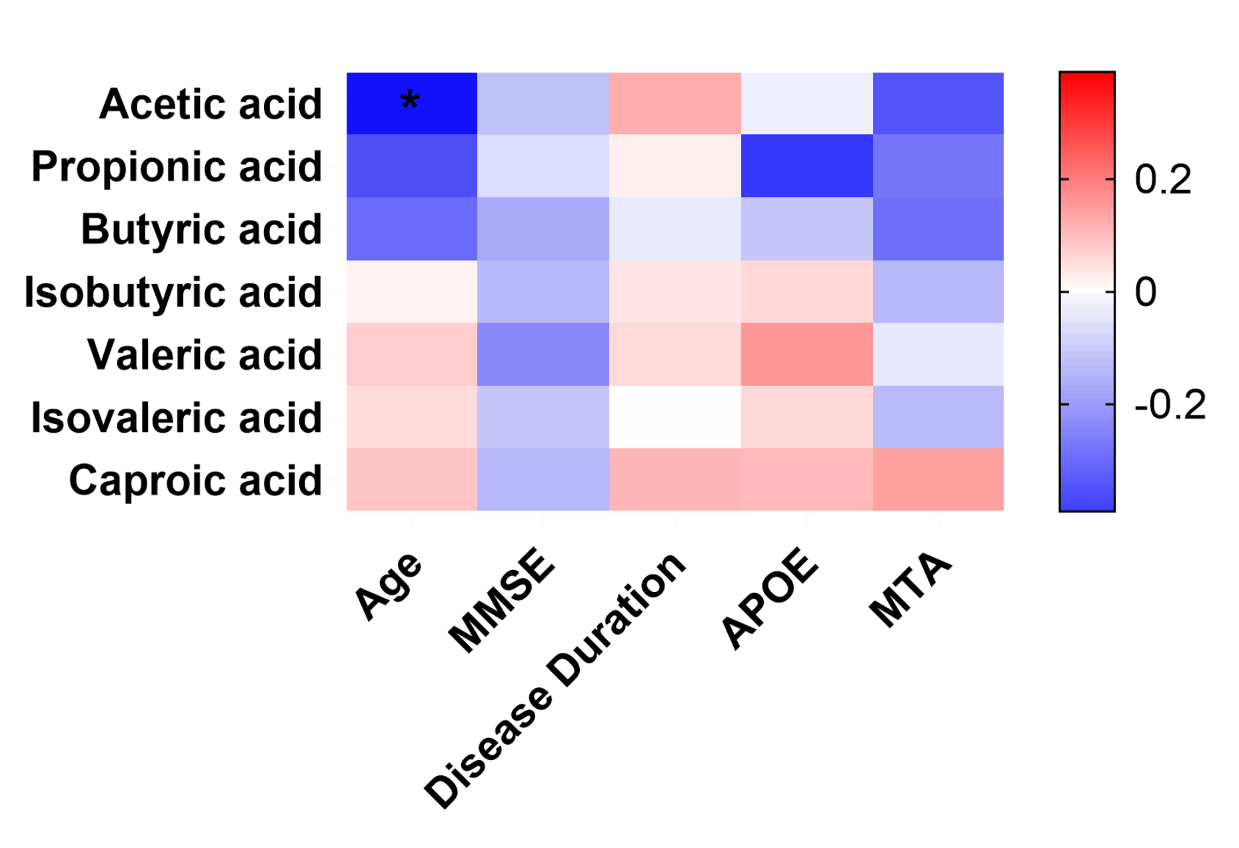


Heat maps of Spearman rank correlation between the altered fecal SCFAs and clinical characteristics including age, cognitive function measured by MMSE, ApoE genotype, disease duration and hippocampus atrophy measured by MTA.

Red means positive correlation and blue means negative.

Abbreviations: SCFAs, short chain fatty acids; MCI, mild cognitive impairment; MMSE, Mini-Mental State Examination; MTA, medial temporal atrophy.

* p<0.05.
